# Comparing the prioritization of items and feature-dimensions in visual working memory

**DOI:** 10.1167/jov.20.8.25

**Published:** 2020-08-25

**Authors:** Jasper E. Hajonides, Freek van Ede, Mark G. Stokes, Anna C. Nobre

**Affiliations:** Oxford Centre for Human Brain Activity, Wellcome Centre for Integrative Neuroimaging, Department of Psychiatry, University of Oxford, UK; Department of Experimental Psychology, University of Oxford, UK

**Keywords:** working memory, attention, feature selection, EEG, retro-cue

## Abstract

Selective attention can be directed not only to external sensory inputs, but also to internal sensory representations held within visual working memory (VWM). To date, this phenomenon has been studied predominantly following retrospective cues directing attention to particular items, or their locations in memory. In addition to item-level attentional prioritization, recent studies have shown that selectively attending to feature dimensions in VWM can also improve memory recall performance. However, no study to date has directly compared item-based and dimension-based attention in VWM, nor their neural bases. Here, we compared the benefits of retrospective cues (retro-cues) that were directed either at a multifeature item or at a feature dimension that was shared between two spatially segregated items. Behavioral results revealed qualitatively similar attentional benefits in both recall accuracy and response time, but also showed that cueing benefits were larger after item cues. Concurrent electroencephalogram measurements further revealed a similar attenuation of posterior alpha oscillations following both item and dimension retro-cues when compared with noninformative, neutral retro-cues. We argue that attention can act flexibly to prioritize the most relevant information—at either the item or the dimension level—to optimize ensuing memory-based task performance, and we discuss the implications of the observed commonalities and differences between item-level and dimension-level prioritization in VWM.

## Introduction

Visual working memory (VWM) provides a means to maintain relevant information independent of continued visual input, to guide adaptive behavior (Baddeley, 1992, 2003). Because VWM has limited capacity and/or resources (Bays & Husain, 2008; Luck & Vogel, 1997; Vogel, Woodman, & Luck, 2001; Zhang & Luck, 2008), it is essential to distribute memory processes efficiently to complete the tasks at hand effectively. Over the past decade, it has become increasingly clear that VWM is more flexible than originally thought. Focused attention continues to prioritize and select contents maintained in VWM as goals and predictions about goals change (Griffin & Nobre, 2003; Kuo, Stokes, & Nobre, 2011; Landman, Spekreijse, & Lamme, 2003; Souza & Oberauer, 2016). To bring about behavioral benefits, attention-related modulatory signals must interact with mnemonic information that is available within VWM. Thus, by studying what forms of attention confer benefits to VWM also provides insight about the format of information held in VWM.

In perceptual attention, research has revealed a multitude of representational formats available for modulation. These include spatial locations, objects, features, semantic associations, time intervals, and likely more (for an overview, see e.g., Nobre, 2018). Whether the same diversity of representational formats is available in VWM is an important and informative question. Information in VWM results from attentional filtering of incoming sensory processing (Vogel, McCollough, & Machizawa, 2005). Thus, the representational information might be kept in an altered, more compact format. For example, it has been suggested that the primary representational unit of VWM involves integrated (feature-bound) items (Luck & Vogel, 1997; Vogel et al., 2001). Under this framework, one might expect that the primary target for attentional selection in VWM should be at the level of individual items. Accordingly, most studies looking at the role of attention in VWM to date have used spatial retro-cues, linked to item-based representations, and have shown clear benefits (e.g., Griffin & Nobre, 2003; Landman et al., 2003; Souza & Oberauer, 2016).

At the same time, recent studies have demonstrated that attention can also facilitate behavior when directed to feature dimensions that are shared among multiple items in VWM (Heuer & Schubö, 2017; Niklaus, Nobre, & Van Ede, 2017; Park, Sy, Hong, & Tong, 2017; Pilling & Barrett, 2016; Sahan, Sheldon, & Postle, 2019; Ye, Hu, Ristaniemi, Gendron, & Liu, 2016; Yu & Shim, 2017). It remains unclear, however, how such effects of dimension-based attention compare with item-based attention in VWM, because no study has directly compared these two forms of attentional facilitation in VWM. Here we directly compare these two types of attentional facilitation.

In addition to comparing item- and dimension-based attention at the level of behavioral performance, we examined their effects on an electrophysiological marker linked to attention in VWM: the attenuation of posterior alpha oscillations. Several studies have revealed that item-based prioritization in VWM is associated with the attenuation of alpha oscillations in posterior brain areas, suggesting modulation of visual areas involved in representing the mnemonic items (Myers, Walther, Wallis, Stokes, & Nobre, 2015; Poch, Capilla, Hinojosa, & Campo, 2017; van Ede, 2018; van Ede, Niklaus, & Nobre, 2017; Wallis, Stokes, Cousijn, Woolrich, & Nobre, 2015; Wolff, Jochim, Akyürek, & Stokes, 2017). It remains unclear whether alpha attenuation also occurs during the attentional prioritization of feature dimensions that are shared across multiple items held in VWM.

In the current study, we therefore compared and contrasted behavioral and neural effects of internal shifts of attention with multifeature items and with single feature dimensions that were shared across multiple items. Through the behavioral data, the aim was to test whether there is a clear primacy of the object-level information in VWM. If the representational format in VWM organizes items as integrated objects, this should also be the primary level at which attention can operate. Accordingly, benefits from item-directing retro-cues should be substantially greater. If, however, attention has similar access to multiple levels of information in VWM, then retro-cueing benefits for feature dimensions and individual items may be similar. By recording EEG and measuring alpha oscillations, we further tested whether a similar alpha modulation occurs when attention is directed to a cued item or to a visual feature dimension that is distributed across multiple items held in VWM.

To address these questions, we used a task in which participants were presented with two Gabor gratings, each of which contained both color and orientation information. On half of the blocks, participants were presented with an item-directing retro-cue and on the other half with a feature-dimension-directing retro-cue. Both blocks contained neutral (uninformative) retro-cues, against which we compared the effects of both types of informative retro-cues. We observed qualitatively comparable retro-cueing effects, although the benefits after item cues were larger. Both effects were accompanied by similar alpha attenuation following the cues, and both item- and dimension-level benefits on behavior were highly correlated across participants.

## Methods

### Participants

The study was approved by the Central University Research Ethics Committee of the University of Oxford and is conducted in accordance with the Declaration of Helsinki. Thirty-two healthy volunteers, 19 female, mean age 28.3 years, range 18 to 35 years, took part. Participants had normal or corrected-to-normal vision and were not color blind. Participants provided written informed consent before participating in the study and were paid £15 per hour. Data from two participants were excluded from analysis, one for terminating the experiment early and the other owing to hardware failure.

### Experimental set-up and stimuli

Participants were seated in front of a 23-inch monitor (1920 × 1080, 100 Hz). Stimuli were generated using Psychophysics Toolbox version 3.0.11 (Brainard, 1997) in MATLAB 2014b (MathWorks, Natick, MA). Head position was set at 90 cm from the monitor, and participants used a chinrest. The stimuli consisted of luminance-defined sinusoidal Gabor gratings generated in MATLAB 2014b. Forty-eight evenly spaced colors were drawn from a circle in CIE L*a*b color space (center at L = 54, a = 18, b = –8, radius = 59). Gratings were presented using one of 48 different orientations (3.75° to 180° in steps of 3.75°) and 48 different colors.

### Task and design

**Figure 1.**
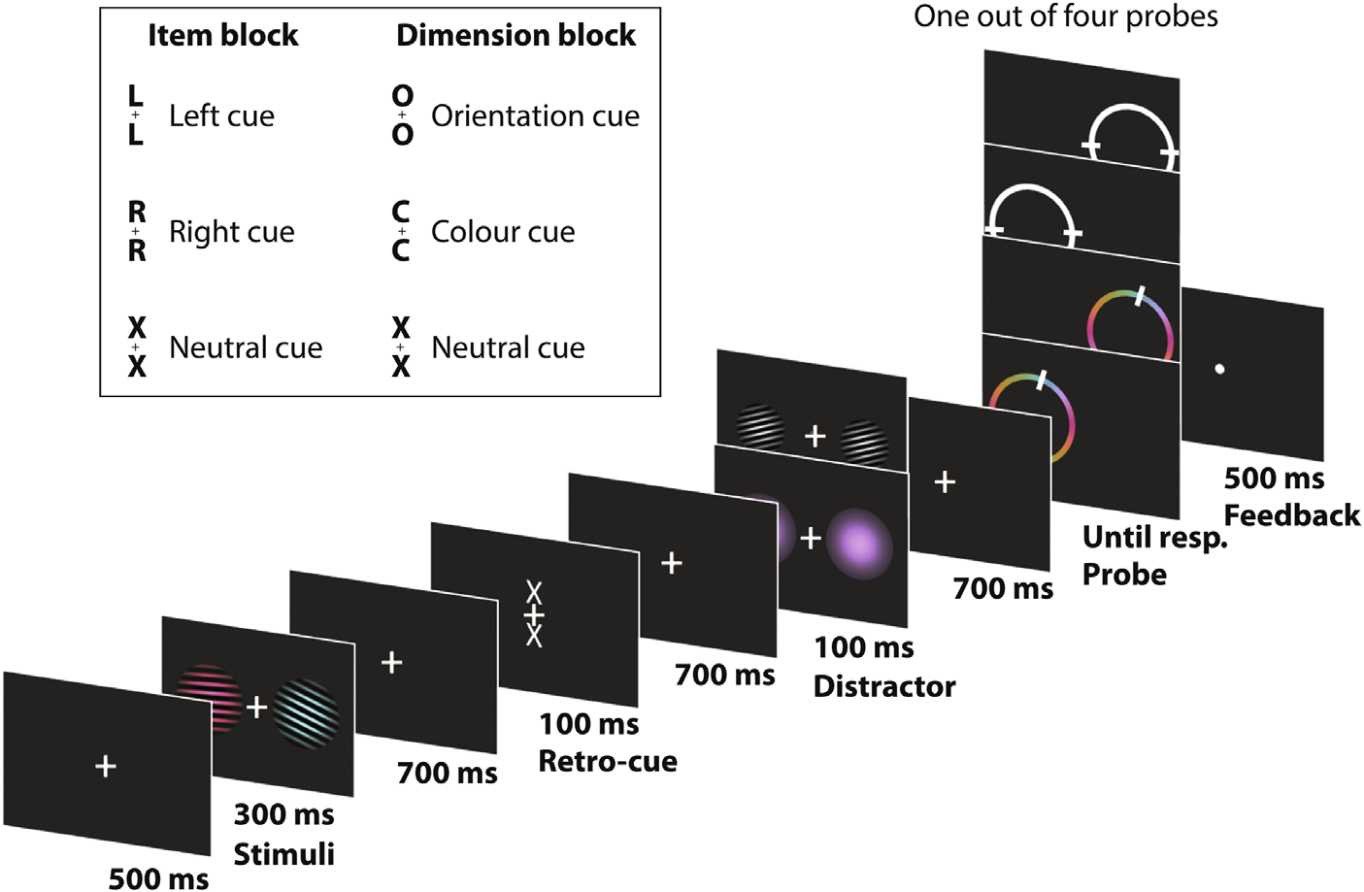
Experimental design with timings. Participants were presented with two colored Gabor gratings to memories. Subsequently, a retro-cue informed participant which item or feature dimension would be probed. After either a color distractor or an orientation distractor with a semi-randomly drawn orientation or color, the probe was presented. Participants adjusted the probe dial to match the feature in memory.

Participants performed 960 trials of a VWM task (Figure 1) in which they were asked to reproduce the color or orientation of one out of two memory items at the end of a memory delay of 2.3 seconds. At the start of each trial, two Gabor stimuli with a radius of 2.2° positioned left and right from fixation (centered 3.1° of visual angle) were presented simultaneously for 300 ms. Participants were instructed to remember the color and the orientation of both items. At the end of the trial, they were probed to report the orientation or the color of one of the items. The to-be-reported feature dimension was indicated with the probe circle that was either a color wheel (color report) or a white wheel (orientation report), whereas the to-be-reported item was indicated by the location of the probe circle (left/right, corresponding with the original location of the probed memory item). Orientation and color values varied independently between the two items, with the constraint that no two equal orientations or colors were presented on the same trial. Colors and orientations were counterbalanced so that each was presented equally often across trials.

Two events occurred during the memory delay: first a retro-cue appeared, which could provide information about the item or feature dimension that would be probed. Second, 700 ms after the retro-cue offset, an irrelevant distractor stimulus was presented that contained either color or orientation information (Figure 1 provides examples). We inserted the distractor with the intention to study the neural response to the distractor as a function of cueing condition. In the end, this aspect did not yield clear results and was therefore omitted for clarity. However, the single-feature nature of the distractor did allow us to investigate whether the congruence of the distractor feature and the probed feature had any effect on recall.

Participants completed 20 blocks in total, each containing 48 trials. On even-numbered blocks, informative retro-cues indicated the location (left [L], or right [R]) of the item that would be probed at the end of the trial, without giving information about what dimension would be probed. On odd-numbered blocks, informative retro-cues indicated the feature dimension to be probed (color, [C], or orientation [O]), without giving information about what item would be probed. We thus cued either a single item that contained two features, or a single feature-dimension that was shared between two items. We note that what we refer to as “feature-dimension” selection is thus distinct from using feature-based cues to orient attention to specific items, for example, as in Heuer and Schubö (2016) and Heuer, Schubö, and Crawford (2016). When informative, the retro-cue was always valid. Both blocks also contained 33% noninformative neutral cues (X) that provided no information about what item or dimension would be probed at the end of the trial. Participants were encouraged to use the informative retro-cues to select the relevant item or feature dimension.

After another fixation period of 700 ms (after the retro-cue), a bilateral distractor was presented for 100 ms. The distractor consisted of either a single color or a black-and-white oriented Gabor grating. Sixteen colors and 16 orientations were used (from 11.25° to 180° in steps of 11.25°). These varied randomly from the orientation/color of the items in the memory array. However, we ensured that the three types of cues (C/O/X or L/R/X) all contained the same range of 16 distractor features. Out of these 16 distractor features, we randomly assigned eight as color features and eight as orientation features.

After another fixation period of 700 ms (after the distractor), the color wheel or a white circle probe appeared with the dial initialized at a random location. To keep the orientation and color recall as similar as possible, we presented the colors at a fixed position on the color wheel. Participants were instructed to respond as accurately as possible by using the *J* and *F* keys to rotate the probe clockwise and counterclockwise, respectively. Participants were instructed to use their left index finger to press the *F* key and the right index finger to press the *J* key. Although there was no explicit time limit for the response time, we logged reaction times as the time between the onset of the probe and the first button press that initiated the dial-up report. Reaction time therefore serves as a proxy for the time it took participants to access the relevant memory information before commencing their reproduction report.

### Behavioral analysis

We computed the error for each trial for each participant by subtracting the target orientation or color (in radians around the color circle in CIE L*a*B space) from the probe response. All error scores were mapped onto a –½π to ½π space. All trials for which the reaction time was more than four standard deviations above a participant's mean decision time were discarded (0.9 ± 0.3%). To calculate the retro-cue benefit, we subtracted the absolute error on cued trials from the absolute error on neutral trials. In all our analyses, we only compared trials of one retro-cueing condition with neutral trials from the same block types (i.e., item blocks or dimension blocks).

A mixture model was fitted separately for each retro-cueing condition and respective neutral condition, modelling target response rate, guess rate, swap responses, and precision to the error data of each subject (Bays, Catalao, & Husain, 2009; Zhang & Luck, 2008). We fitted the mixture model separately for every subject, color, or orientation recall, spatial or dimension retro-cues, and informative or neutral retro-cues. Estimating the mixture-model parameters allowed for estimation of different components that contribute the overall error; we estimated the fidelity of the representation independently of the guess and swap rate. We used the mixture model made available by Bays et al. (2009).

When comparing more than two conditions, we applied a repeated-measures analysis of variance and report η^2^ as a measure of effect size. When evaluating retro-cueing benefits, we applied dependent samples *t* test, comparing informative vs neutral cues, as well as the cueing effects between item and dimension retro-cues. When the assumption of normality was violated we instead applied a Wilcoxon signed-rank test. We report Cohen's d as a measure of effect size for parametric tests and matched rank biserial correlation for nonparametric effect size. For evaluation we used two-sided tests with a critical alpha value of 0.05.

### Electroencephalogram (EEG) acquisition

EEG data were collected using Synamps amplifiers and Neuroscan software (Compumedics). We used a 61 Ag/AgCl sintered electrodes (EasyCap, Herrsching, Germany), laid out according to the international 10–10 system, with mastoids behind the left and right ear. The left mastoid was used as an active reference during the recordings. Offline, an average mastoids reference was derived using the left and right mastoids. The ground electrode was placed on the left arm above the elbow. Horizontal electrooculogram (EOG) was measured using lateral electrodes next to both eyes and vertical EOG was measured above and below the left eye. Data were sampled at 1000 Hz and stored for subsequent analysis.

### EEG preprocessing

Data were imported into MATLAB 2017a using *pop_loadcurry()* and further analyzed using Fieldtrip (Oostenveld, Fries, Maris, & Schoffelen, 2011) and the OHBA Software Library (OSL; https://ohba-analysis.github.io/). Analysis started by cutting out the epochs between 100 ms before and 2200 ms after retro-cue onset *ft_redefinetrial()* followed by re-referencing the data to the average of the mastoids *ft_preprocessing()*. EEG data were down-sampled to 200 Hz to decrease computational demands and storage space *ft_resampledata()*.

Next, EEG data were further de-noised using independent component analysis *ft_componentanalysis()* applying the FastICA algorithm (Hyvärinen, 1999) to all EEG sensors. Independent component analysis separates the EEG signal into non-Gaussian subcomponents of the data that are statistically independent from one another. Spatial components strongly correlated (r > 0.4) with EOG channels were removed from the EEG data. We set out to remove trials on which participants blinked during the window of 100 ms prior up to 200 ms after retro-cue presentation. After baselining the horizontal EOG signal at –300 to –100 ms trials on which horizontal EOG voltage surpassed 200 µV (approximately one-half of the maximum voltage evoked by a typical blink) were flagged and later removed from EEG and behavioral analyses (0.446 ± 1.23%). Subsequently, we removed epochs based on within-trial variance of the broadband signal at a 0.05 significance threshold using a generalized ESD test (Rosner, 1983; implemented in OSL) and discarded 2.48 ± 2.18% of the trials.

### Time–frequency processing

Time–frequency decomposition of the EEG signal was done using ft_freqanalysis. Spectral power between 2 and 50 Hz was computed on Hanning-tapered data using a short-time Fourier transform, with a 300-ms sliding time window that was advanced in steps of 15 ms. We zoom in on modulations in posterior alpha oscillations, by averaging the time-frequency plots for the left cues (P1, P3, P5, P7, PO3, PO7, and O1) and right (P2, P4, P6, P8, PO4, PO8, and O2) posterior electrodes and calculating the normalized differences in power falling between informative and neutral retro-cues ([informative—neutral]/[informative + neutral] × 100). We did this separately for left and right item retro-cues, and for color and orientation dimension retro-cues. Using the same quantification, we also compared alpha power for contralateral versus ipsilateral electrodes after item, and alpha for color versus orientation after a dimension cue. For statistical evaluation, we applied a two-sided cluster-based permutation analysis (Maris & Oostenveld, 2007) with 5,000 permutations at an evaluation threshold of 0.05.

To characterize the onset of alpha attenuation after the retro-cue, we extracted the time course of 7 to 12 Hz power modulation (in the specified informative vs. neutral cue contrast) and focused on the 0 to 1,000 ms period after retro-cue onset. On these data, we then identified the earliest timepoint in which the power modulation reached half of its minimal value for each condition. This latency was used as a measure to compare neural modulation by dimension retro-cues and item retro-cues.

To depict the topography of the power modulations analyzed in the predefined set of posterior electrodes (depicted in Figure 4), we calculated the relevant contrast for each electrode and averaged over the time-frequency window of 400 to 800 ms and 7 to 12 Hz. In addition, to focus on alpha lateralization, we contrasted activity in electrodes left posterior electrodes (O1, PO7, PO3, P7, P5, P3, and P1) and right posterior electrodes (O2, PO8, PO4, P8, P6, P4, and P2), contralateral versus ipsilateral to the cued item after informative item retro-cues. To visualize the topography of the difference between electrodes contra- and ipsilateral to the item cue, we mirrored the left and right electrodes onto one side. We then subtracted electrodes that would be ipsilateral relative to the cued item from the electrodes that would be contralateral relative to the cued item.

Topographies were intended solely to portray the nature of the modulation and were not subjected to further statistical testing.

## Results

**Figure 2.**
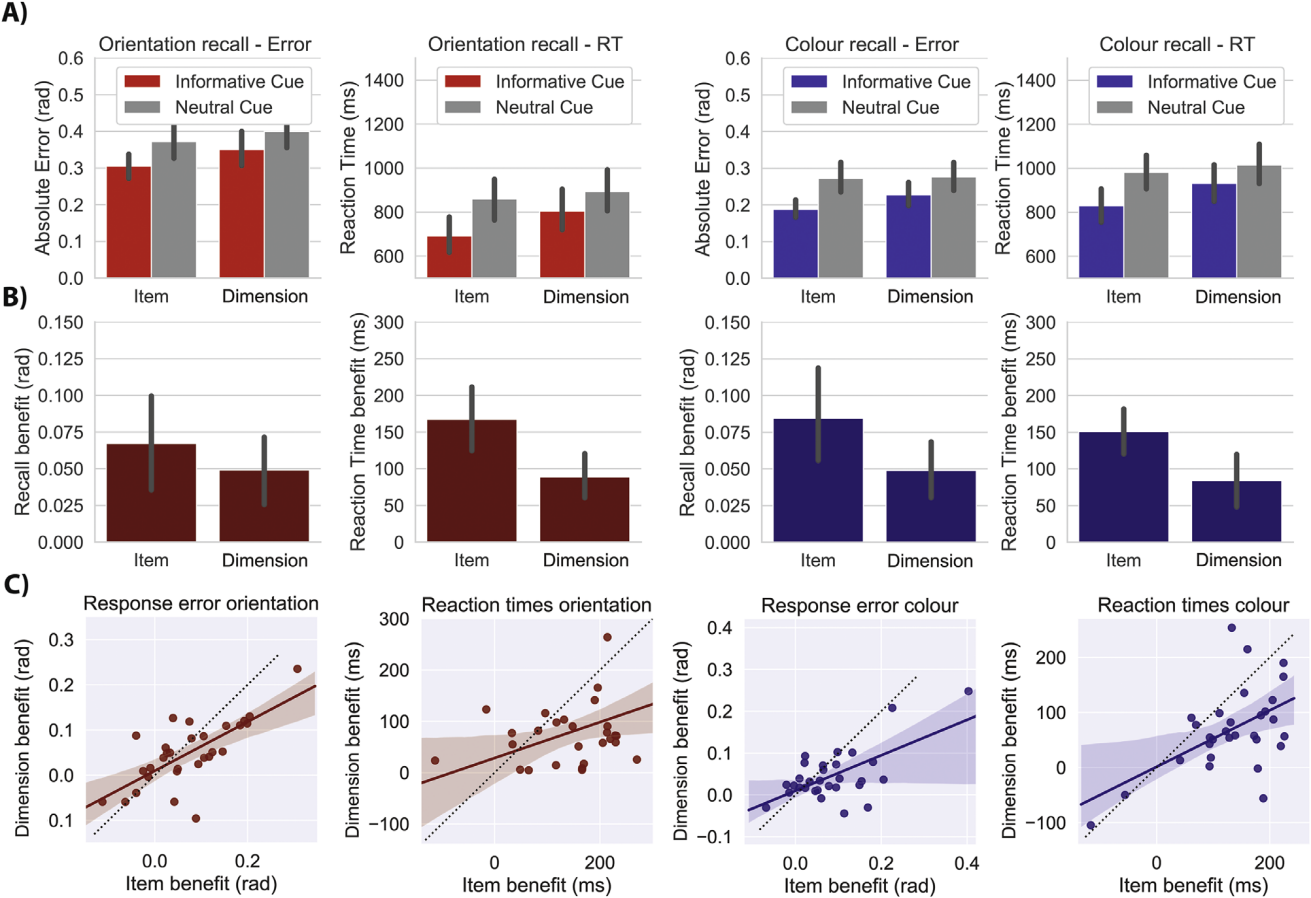
Performance benefits of item and feature-dimension retro cueing. (A) The four columns show absolute error and reaction times for trials with an informative cue or a neutral cue. Trials in which orientation was probed are displayed in *red* while color-probe trials are displayed in *blue*. Each panel shows the data separately for item retro-cue blocks and dimension retro-cue blocks. (B) Behavioral benefit of retro-cues. Subtracting the mean absolute error on trials with an informative cue from the neutral trials gives the performance benefit of the retro-cue, here expressed as positive values. Orientation benefit is depicted in *dark red* and color benefit in *dark blue*. (C) Correlations across participants between the item and dimension retro-cue benefits in shown in B. Error bars show 95% confidence intervals.

Figure 2A shows behavioral performance as a function of experimental condition (collapsed over distractor type, because this did not yield consistent results, as discussed elsewhere in this article). To analyze the effects of item and feature-dimension retro-cues, we quantified retro-cueing benefits as the difference between the trials with informative and neutral retro-cues (Figure 2B).

To formally quantify the effects of retro-cue informativeness (valid or neutral) and retro-cue block type (item retro-cue block or dimension retro-cue block) we used a 2 × 2 repeated-measures analysis of variance. We ran this separately for response time and response error, and separately for both color and orientation recall reports. We observed a significant main effect of retro-cue informativeness, with better performance following informative versus neutral retro-cues on all four dependent variables: orientation error, *F*(1, 29) = 18.888, *p* < 0.001, η^2^ = 0.394; color error, *F*(1, 29) = 26.387, *p* < 0.001, η^2^ = 0.476; orientation response time, *F*(1, 29) = 67.27, *p* < 0.001, η^2^ = 0.699; and color response time, *F*(1, 29) = 65.15, *p* < 0.001, η^2^ = 0.692. At the same time, we found that the behavioral benefits of retro-cue informativeness were larger in item retro-cue block than in dimension retro-cue block, yielding a significant interaction for color error, *F*(1, 29) = 9.065, *p* = 0.005, η^2^ = 0.238; orientation response time, *F*(1, 29) = 19.64, *p* < 0.001, η^2^ = 0.404; and color response time, *F*(1, 29) = 21.00, *p* < 0.001, η^2^ = 0.420. Although we found the same trend, this did not reach significance for orientation error, *F*(1, 29) = 3.750, *p* = 0.063, η^2^ = 0.115. Finally, in line with the greater benefit of item retro-cues, we also found a significant main effect of block type, constituted by better performance in item retro-cue blocks for all four dependent variables: orientation error, *F*(1, 29) = 39.634, *p* < 0.001, η^2^ = 0.577; color error, *F*_1,29_ = 8.343, *p* = 0.007, η^2^ = 0.223; orientation response time, *F*_1,29_ = 50.51, *p* < 0.001, η^2^ = 0.635; and color response time, *F*(1, 29) = 32.08, *p* < 0.001, η^2^ = 0.525.

We also considered the third factor, namely, distractor congruence (i.e., when the distractor contained the same or the other feature dimension as the to-be-recalled memory dimension), but found no systematic effects of distractor congruence across our four dependent variables, nor interactions with the factors of interest (Supplementary Table 1)*.*

We describe in greater detail the item and dimension retro-cueing effects of interest, in accordance with the data presented in Figure 2.

For orientation recall reports, participants significantly benefitted from item retro-cues. They had smaller errors, *t*_29_ = 4.235, *p* < 0.001, d = 0.773, and responded faster, *t*_29_ = 7.854, *p* < 0.001, d = 1.434*,* compared with trials with neutral retro-cues in the same block types. Similarly, orientation reports benefitted significantly from dimension cues in both reproduction error, *t*_29_ = 3.748, *p* = 0.001, d = 0.684, and response onset time, *t*_29_ = 6.302, *p* < 0.001, d = 1.151, compared with neutral trials within the dimension retro-cueing blocks. Item cues conferred numerically larger benefits than dimension cues. The difference was not statistically significant for error, 0.023 rad., 48%, *t*_29_ = 1.936, *p* = 0.063, d = .354, but reached significance for reaction times, 79 ms, 94%, *t*_29_ = 4.431, *p* < 0.001; d = 0.809.

The same pattern of results was found for the error and reaction times in the color recall trials: color reports benefitted from both item cues, *t*_29_ = 5.060, *p* < 0.001, d = 0.924, and dimension cues, *t*_29_ = 4.069, *p* < 0.001, d = 0.743, and responses were also faster for item cues, *t*_29_ = 9.097, *p* < 0.001, d = 1.661, and dimension cues, *t*_29_ = 4.951, *p* < 0.001, d = 0.904, compared with their respective neutral trials. For color reports, we also found greater benefits of item retro-cues compared with dimension retro-cues for both error, 0.039 rad., 86%, *t*_29_ = 3.011, *p* = 0.005, d = 0.550, and reaction time, 63 ms, 91%, *t*_29_ = 4.583, *p* < 0.001, d = 0.837.

The benefits of item-based and dimension-based retro-cueing showed strong positive correlations across individuals for both color and orientation reports (Figure 2C). For orientation reports, we found significant correlations between retro-cueing benefits following item cues and dimension cues for both error, *r* = .709, *p* < 0.001, and reaction time, *r* = .539, *p* = 0.002,. Likewise, for color reports, we found significant correlations between retro-cueing benefits following item cues and dimension cues for both error, *r* = .697, *p* < 0.001, and reaction time, *r* = .596, *p* < 0.001. Thus, participants who benefitted most from item retro-cues also benefitted most from dimension retro-cues.

### Mixture modelling

**Figure 3.**
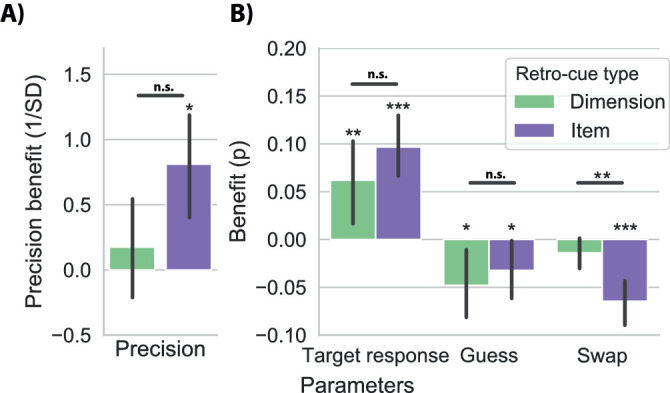
Cueing effects on mixture modelling parameters. (A) Mixture-model estimates for the cueing effects on (A) precision, (B) target response, guess rate, and swap rate, for trials where item retro-cues or dimension retro-cues were presented compared with neutral trials. Hence, valid retro-cues positively influenced target response proportions and negatively influenced guess rate and swap rate. The asterisks indicate significant differences of, respectively, item or dimension benefits from zero (i.e., benefits following informative versus neutral cues). Asterisks above horizonal lines indicate significant differences between item and dimension retro-cueing benefits. Error bars indicate 95% confidence intervals. n.s: *p* > .05, * *p* < 0.05, ** *p* < 0.01, *** *p* < 0.001.

In addition to the raw behavioral scores, we also modelled sources of error using a mixture model (Figure 3AB; Bays et al., 2009). We modelled four components 1) precision, characterized by width (1/STD) of the target centered response distribution, 2) proportion of target responses modelled by the gaussian centered around the target, 3) proportion of random responses characterized by the height of the uniform response distribution, and 4) proportion of responses to the noncued feature of the same dimension as the cued dimension (nontarget report or swap errors). Figure 3A and B shows the retro-cueing effects (informative vs. neutral) on each of these four parameters, separately for item and dimension cues (collapsed over color and orientation reports, after fitting the model for each dimension separately; Supplementary Figure 1 provides mixture model parameters separated for color and orientation reports). As depicted in Figure 3A and B informative (vs. neutral) retro-cues significantly increased precision for item retro-cues, item: *t*_29_ = 2.736, *p* = 0.011, d = 0.500, although this did not reach significance for dimension retro-cues: *t*_29_ = 0.578, *p* = 0.568, d = 0.105. At the same time, both item and dimension retro-cues increased target response rates, item: *t*_29_ = 5.595, *p* < 0.001, d = 1.022, dimension: Z_29_ = 382, *p* = 0.001, r_rb_ = 0.643, and decreased guess rates, item: *t*_29_ = –2.131, *p* = 0.042, d = –0.389, dimension: Z_29_ = 78, *p* < 0.001, r_rb_ = –0.665, and item retro-cues further decreased swap rate, item: Z_29_ = 21, *p* < 0.001, r_rb_ = –0.910, dimension: Z_29_ = 137, *p* = 0.080, r_rb_ = –0.368.

Direct comparisons between item and dimension retro-cue benefits showed a significantly greater reduction in the rate of swap errors by item retro-cues relative to dimension retro-cues, Wilcoxon signed-rank test, Z_29_ = 95, *p* = 0.002, r_rb_ = 0.591 (Figure 3A and B). Effects for the other three parameters were not statistically different between item and dimension retro-cues, all *p* > 0.10.

### Alpha attenuation after feature and item retro-cues

**Figure 4.**
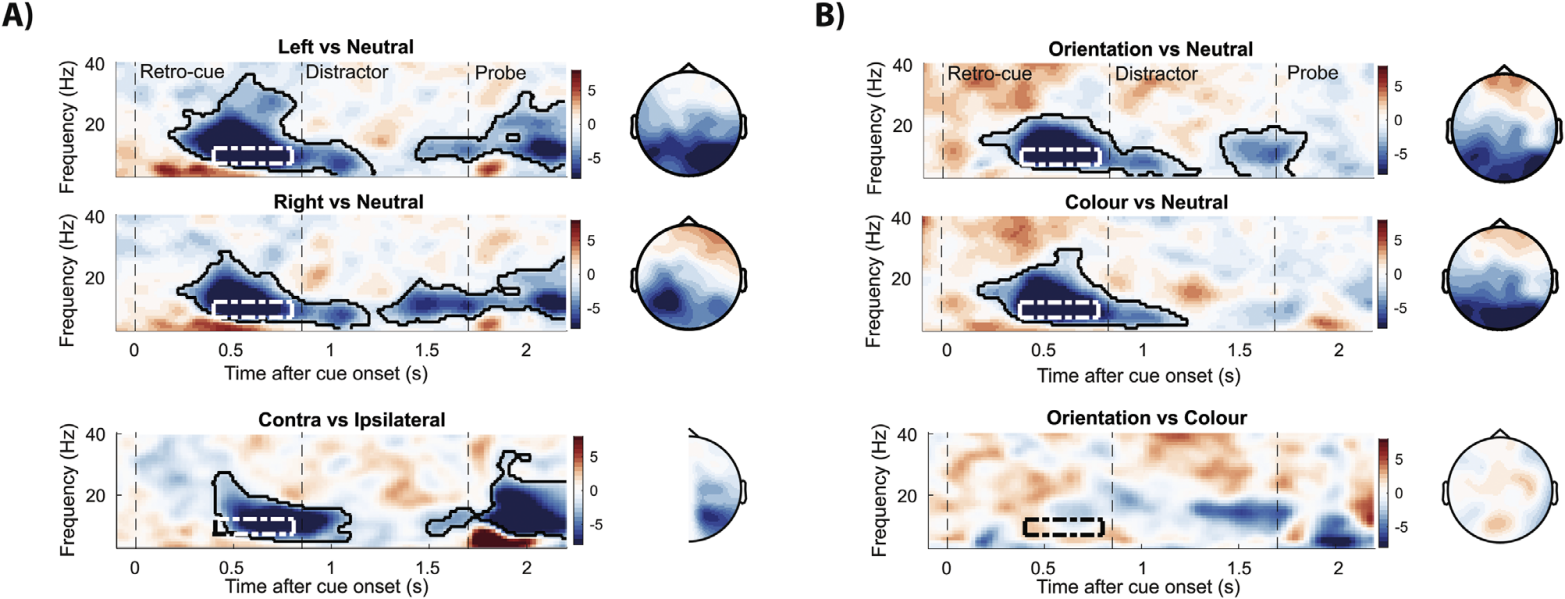
Induced neural EEG responses to the left and right item retro-cues and to color and orientation dimension retro-cues. (A) Time–frequency representation of the difference between left/right cued trials versus neutral trials in item–cue blocks in left (P1, P3, P5, P7, PO3, PO7, and O2) and right (P2, P4, P6, P8, PO4, PO8, and O2) electrodes. The bottom panel depicts the time-frequency map of the difference between contralateral and ipsilateral responses calculated across left and right posterior electrodes, as described in the Methods. Topographies show the associated differences in alpha power (7–12 Hz) in the 400- to 800-ms window (indicated in the *dotted* *line boxes* in all time–frequency maps). For the contralateral vs ipsilateral topography (*bottom*), we projected the relevant contrast values into the right electrode of each electrode pair. (B) Same representations as outlined above but here we compare color and orientation with their respective neutral trials or with one another, in left and right electrodes. Highlighted areas with the black solid outline indicate significant clusters (permutation test, *n* = 30, cluster-forming threshold *p* < 0.05, corrected significance threshold *p* < 0.05).

Figure 4 shows the time- and frequency-resolved modulations in spectral EEG power in posterior electrodes after item and dimension retro-cues, expressed as a difference from the neutral retro-cueing condition (neutral retro-cue minus informative retro-cue). After both item retro-cues and dimension retro-cues, we observe an attenuation of alpha power starting at around 400 ms after presentation of the retro-cue (clusters all conditions *p* < 0.001). The alpha attenuation in trials with informative retro-cues reemerges after the distractor onset, in the window just prior to the probe. To reveal the spatial layout of the significant clusters, we visualized the EEG topographies of the alpha-band power at 400 to 800 ms after the retro-cue. Similar topographies were associated with the later alpha modulation after the distractor and with the early modulation in the higher 13- to 30-Hz band (topographies not depicted).

In addition to this global effect when comparing informative to neutral retro-cues, we also evaluated the specific difference between left and right item cues (from the perspective of electrodes contra- and ipsilateral to the cued item), and between color and orientation (Figure 4, bottom row). In line with several prior studies (Myers et al., 2015; Poch et al., 2017; van Ede et al., 2017; Wallis et al., 2015; Wolff et al., 2017), following item cues, alpha attenuation was most pronounced contralateral to the memorized location of the cued item. In contrast, following dimension retro-cues no clear differences were observed between color and orientation cues, which directed attention to a single feature dimension that was shared between the left and right items. Finally, we found that the alpha attenuation had very similar latencies following item-directing and dimension-directing retro-cues, t_29_ = 1.273, *p* = 0.213 (Supplementary Figure 2). Further exploring the data, we also analyzed event-related potentials (ERP) following retro-cues to investigate ERP components linked to attentional selection in working memory. We found clear selection-related potentials following retro-cues that afforded item-level selection (Supplementary Figure 3). At the same time, however, we found no clear lateralization of ERPs after item cues, nor significant ERP modulations associated with attentional selection following dimension cues.

## Discussion

We demonstrate that both item-based and dimension-based attentional prioritization during VWM maintenance decreases recall error and speeds response initiation times following the probe. Hence, we support the finding that selective attention can retrospectively prioritize not only items (Griffin & Nobre, 2003; Kuo et al., 2011; Landman et al., 2003; Souza & Oberauer, 2016), but also feature dimensions maintained in VWM (Heuer & Schubö, 2017; Niklaus et al., 2017; Park et al., 2017; Sahan et al., 2019; Ye et al., 2016; Yu & Shim, 2017). We thereby replicated a feature prioritization effect in VWM for more than one location (unlike Makovski & Jiang, 2007; but see also Heuer & Schubö, 2016; Lepsien, Thornton, & Nobre, 2011; Matsukura & Vecera, 2015). Building on this work, our experimental design uniquely allowed us to compare the magnitudes of both types of behavioral retro-cue benefits within a single experiment, and to correlate their strengths across participants. Although the item benefit was greater than the dimension benefit, both were highly robust. They were each evident across both color and orientation reports and in both recall accuracy and response initiation times. Moreover, we found strong correlations between the benefits that followed item and dimension cues, and qualitatively similar neural modulations, which suggest that the two types of retro-cueing benefits may share at least overlapping (although not necessarily equivalent) attentional mechanisms.

The notion that both retro-cueing types yield behavioral benefits that are qualitatively similar was further supported by the similar retro-cueing effects on guess rate and target response rate parameters estimated by the mixture model. At the same time, we observed that only item cues significantly enhanced precision and decreased the probability of swaps (nontarget responses), with the latter being the only parameter that also differed significantly between item and dimension retro-cue benefits. This difference is likely explained by the fact that swaps are calculated between items (within a single dimension). Provided that feature-cues always concerned one dimension, shared across both items, they may have helped to upregulate the relevant feature dimension, but not to separate the two spatially segregated items and thereby to reduce swap rates (in contrast with item cues that directly targeted the relevant item from the two memorized items). The mixture modelling, therefore, enables the separation of error into its different sources, such as swap rate and guess rate. The overall error should reflect the aggregate of all mixture modelling components.

In a strict account in which the primary unit of VWM is integrated items (Luck & Vogel, 1997; Vogel et al., 2001), one may predict that attention in VWM will primarily operate at the level of items, leaving little room for attentional facilitation of specific features that are shared among items. Alternatively, if VWM consists of a hierarchy of representations, with both item-level and dimension-level representations (Bays, Wu, & Husain, 2011; Brady, Konkle, & Alvarez, 2011; Fougnie & Alvarez, 2011; Töllner, Conci, Müller, & Mazza, 2016; Töllner, Mink, & Müller, 2015); then one may expect that attention can operate similarly at distinct levels, depending on the nature of the task at hand. Our data are in line with a mixture of both scenarios—showing that attention can operate qualitatively similarly at both item and dimension levels, while also revealing an additional benefit when attention is directed at two dimensions of a single item (following item cues), compared with a single feature dimension across two items (following dimension cues).

At the same time, we note that attentional benefits in behavioral performance in VWM tasks need not only reflect changes in the quality of representational information. Factors related to prospective task preparation may also contribute (González-García, Formica, Liefooghe, & Brass, 2020; Myers, Stokes, & Nobre, 2017; van Ede, Chekroud, Stokes, & Nobre, 2019). Therefore, although our data provide clear evidence for the benefit of dimension retro-cues—which is qualitatively similar to, and correlated with, the benefit following item cues—it remains possible that at least part of these benefits are due to factors other than a change in the underlying mnemonic representation (and this holds for both item and dimension retro-cueing benefits).

In addition to the behavioral performance data, we also observed commonalities in the neural modulation following item and dimension cues; both cases showing robust alpha attenuation over posterior electrodes, arising around the same time, with a similar magnitude. The neural responses therefore provide important relevant complementary data to our behavioral performance data. We observe more direct evidence for an early modulation in posterior (putatively visual) brain areas following both types of retro-cues; compatible with a modulation at the level of the memorized visual representations. This finding may reflect reweighting of sensory input in order to optimize upcoming memory-guided behavior (Myers, Stokes, & Nobre 2017). However, despite the similar appearance of this modulation following item- and feature-dimension retro-cues, it remains possible that the neural signals we measure reflect the engagement of distinct, but overlapping, brain areas and neural computations (Heuer, Schubö, & Crawford 2016), just like the recruitment of different neural substrates for item-selection based on spatial versus feature cues (Heuer & Schubö 2016). However, because we used visual retro-cues, we cannot fully rule out the possibility that at least part of this modulation may be driven by differential visual processing of informative versus a neutral retro-cues per se, although we note how our neutral retro-cues were designed to be similar to our informative retro-cues, ruling out more obvious differences owing to bottom-up visual features such as retro-cue size and saliency. One important difference between the two types of selection is that spatial orienting is inherently linked to item selection and not to dimension selection. Items occupied distinct locations and each dimension was always shared between two segregated items. In future studies, it will also be informative to equate spatial factors when comparing item and dimension selection, such as by using multifeature items that occupy the same spatial location (e.g., overlapping colored moving dot clouds).

In conclusion, retro-cueing studies have typically shown that internally directed attention can prioritize a subset of mnemonic representations (Griffin & Nobre, 2003; Rerko, Souza, & Oberauer, 2014; Van Moorselaar, Olivers, Theeuwes, Lamme, Victor, & Sligte, 2015). These representations are typically thought of as integrated item of features bound together into a discrete mnemonic item (Luck & Vogel, 1997; Vogel et al., 2001). Our results show that attention can also effectively be directed to specific visual dimensions that are shared across multiple items in memory—and for the first time reveal that such dimension cues yield qualitatively similar (albeit weaker) behavioral benefits and neural modulations or latency, as do item cues, and that item and dimension cueing benefits are correlated across individuals. We argue that retro-cues help place memorized visual stimuli into a goal-oriented format, such that relevant information at both the item and the dimension level can be optimized for upcoming task performance.

## Supplementary Material

Supplement 1
