# Supplementary material for: Comparing the prioritization of items and feature-dimensions in visual working memory
Source: J Vis. 2020 Aug 25;20(8):25. doi: 10.1167/jov.20.8.25 (PMC7453048; doi:10.1167/jov.20.8.25)
Supplement: Supplement 1 [file jovi-20-8-25_s001.pdf]

Supplementary materials

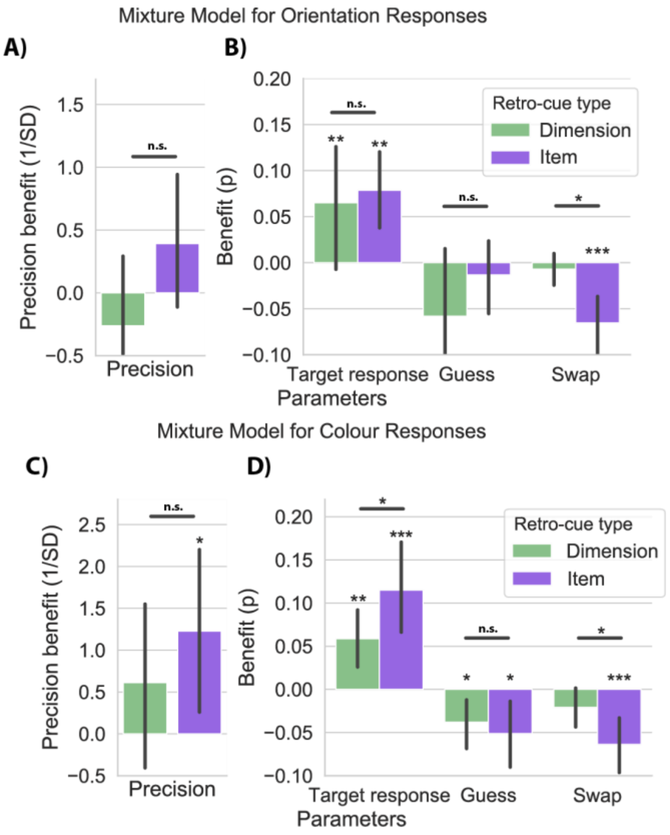

Supplementary Figure 1. Mixture modelling parameters for colour and orientation. Mixture-model estimates for the benefit of orientation recall in A-B) and colour recall in C-D). Mixture model parameters include precision (A, C), target response, guess rate, and swap rate (B, D) for trials where item retro-cues or feature retro-cues were presented compared to neutral trials. Black asterisks indicate significant differences between item – and feature retro-cueing benefits. Asterisks above bars represent significance of a two-sided t-test of the model parameter benefit against zero. Error bars indicate 95% confidence intervals. * $p < .05$, ** $p < .01$, *** $p < .001$.

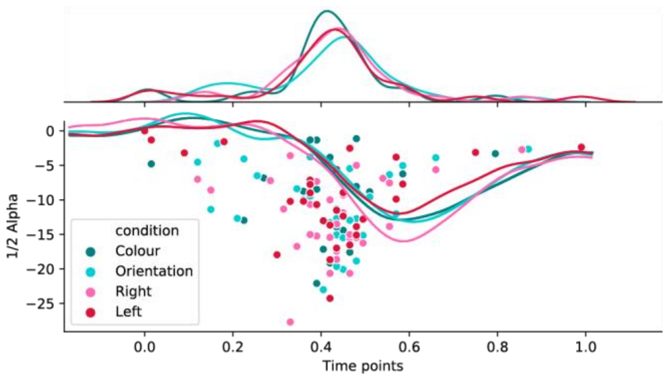

Supplementary Figure 2. Onset times of the alpha attenuation after the retro-cue. The first time point where the alpha power (neutral – informative cue) reached half of its minimum value in the interval from 0 to 1000 ms after the retro-cue onset was taken as the alpha attenuation latency. The dots represent alpha attenuation latency times for individual subjects and different cueing conditions with a density plot on the top

showing the density of the dots along the x-axis for each condition. The line plot illustrates the average alpha power for each condition after subtracting their relative neutral retro-cues.

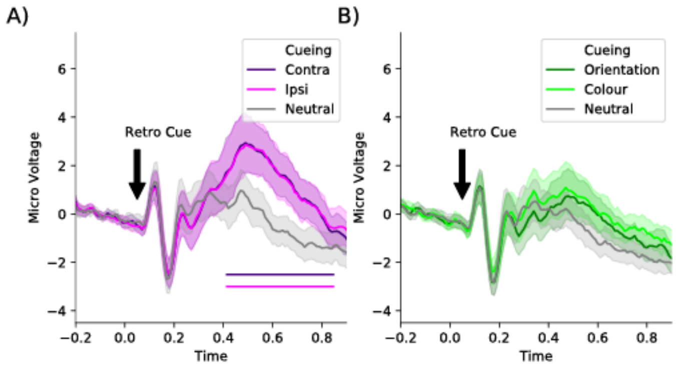

Supplementary Figure 3. Event-related potentials following informative and neutral retro-cues. Event-related potentials in left (P1, P3, P5, P7, PO3, PO7, O2) and right (P2, P4, P6, P8, PO4, PO8, O2) posterior electrodes following informative and neutral retro-cues. We plotted A) the average voltages for electrodes contralateral and ipsilateral to the cued item following item-selection cues as well as B) the average evoked potentials collapsed across posterior electrodes following colour or orientation dimension-selection cues. Grey lines show responses to neural (uninformative) retro-cues for comparison. Error bars show 95% confidence intervals. Horizontal lines show clusters of significant differences between informative cues tested against neutral cues (t_{29} threshold of 2.045, 10000 permutations).

Supplementary table 1. Main effects and interactions for the congruence or incongruence of the distractor with the probed memory feature, tested with a 2 x 2 x 2 rmANOVA with the factors distractor congruence, cue informativeness, and block type, separately for each of our four dependent variables.

B) Within Subjects Effects for **Orientation recall: Error**

	Sum of Squares	df	Mean Square	F	p	η^2
Distractor	0.022	1	0.022	5.515	0.026	0.160
Residual	0.116	29	0.004			
Retro-cue type ✻ Distractor	7.879e -4	1	7.879e -4	0.256	0.617	0.009
Residual	0.089	29	0.003			
Informativeness ✻ Distractor	4.938e -4	1	4.938e -4	0.142	0.709	0.005
Residual	0.101	29	0.003			
Retro-cue type ✻ Informativeness ✻ Distractor	5.667e -5	1	5.667e -5	0.026	0.872	0.001
Residual	0.063	29	0.002			

B) Within Subjects Effects for **Colour recall: Error**

	Sum of Squares	df	Mean Square	F	p	η^2
Distractor	7.333e -4	1	7.333e -4	0.286	0.597	0.010
Residual	0.074	29	0.003			
Retro-cue type ✻ Distractor	0.001	1	0.001	1.290	0.265	0.043
Residual	0.023	29	7.788e -4			
Informativeness ✻ Distractor	0.001	1	0.001	0.643	0.429	0.022
Residual	0.050	29	0.002			
Retro-cue type ✻ Informativeness ✻ Distractor	2.928e -4	1	2.928e -4	0.295	0.591	0.010
Residual	0.029	29	9.912e -4			

C) Within Subjects Effects for **Orientation recall: RT**

	Sum of Squares	df	Mean Square	F	p	η^2
Distractor	8427	1	8427	1.939	0.174	0.063
Residual	126029	29	4346			
Retro-cue type ✻ Distractor	6784	1	6784	2.556	0.121	0.081
Residual	76971	29	2654			
Informativeness ✻ Distractor	1807	1	1807	0.549	0.465	0.019
Residual	95526	29	3294			
Retro-cue type ✻ Informativeness ✻ Distractor	2004	1	2004	0.652	0.426	0.022
Residual	89135	29	3074			

D) Within Subjects Effects for **Colour recall: RT**

	Sum of Squares	df	Mean Square	F	p	η^2
Distractor	6517.18	1	6517.18	0.629	0.434	0.021
Residual	300276.91	29	10354.38			
Retro-cue type ✻ Distractor	339.93	1	339.93	0.119	0.733	0.004
Residual	83020.69	29	2862.78			
Informativeness ✻ Distractor	1762.09	1	1762.09	0.698	0.410	0.024
Residual	73178.64	29	2523.40			
Retro-cue type ✻ Informativeness ✻ Distractor	54.09	1	54.09	0.023	0.879	0.001
Residual	66963.22	29	2309.08			
